# Evaluation of a mouthrinse containing guava leaf extract as part of comprehensive oral care regimen- a randomized placebo-controlled clinical trial

**DOI:** 10.1186/s12906-019-2745-8

**Published:** 2019-11-21

**Authors:** Namratha Nayak, Jothi Varghese, Seema Shetty, Vinutha Bhat, Tejal Durgekar, Richard Lobo, Usha Y. Nayak, Vishwanath U

**Affiliations:** 1 Department of Periodontology, Manipal College of Dental Sciences, Manipal Academy of Higher Education, Manipal, Karnataka India; 2 Department of Microbiology, Kasturba Medical College, Manipal Academy of Higher Education, Manipal, Karnataka India; 3 Department of Biochemistry, Kasturba Medical College, Manipal Academy of Higher Education, Manipal, Karnataka India; 4 Department of Pharmacognosy, Manipal College of Pharmaceutical Sciences, Manipal Academy of Higher Education, Manipal, Karnataka India; 5 Department of Pharmaceutics, Manipal College of Pharmaceutical Sciences, Manipal Academy of Higher Education, Manipal, Karnataka India; 6 Department of Biotechnology and Microbiology, Sri Dharmasthala Manjunatheshwara Centre for Research in Ayurveda and Allied Sciences, Kuthpady, Udupi, Karnataka India

**Keywords:** Chronic gingivitis, Guava mouth rinse, Chlorhexidine mouth rinse, Microbial CFU counts, Antioxidant levels

## Abstract

**Background:**

The control of biofilm adherence on tooth surface has always been the keystone of periodontal therapeutic systems. However, prevalence of gingivitis suggest inadequacy of self-performed oral hygiene measures and need for adjunctive aid for mechanical plaque control. Oral rinses containing chlorhexidine, has been widely used however, with certain limitations. Herbal products have been used widely reflecting its action as alternative and complementary remedy. Hence, the purpose of the present study was to evaluate the antimicrobial and antioxidant efficacy of a Guava leaf extract based mouthrinse in patients with chronic generalized gingivitis as an adjunct to oral prophylaxis.

**Methods:**

Sixty subjects (*n* = 20) in compliance with the inclusion criteria were randomly assigned to one of the 3 study groups i.e. Group A- 0.15%Guava mouth rinse, Group B- 0.2% Chlorhexidine (CHX) mouth rinse, Group C- Distilled water (placebo). All the participants received professional oral prophylaxis and were dispensed with experimental mouth rinses and instructed to use for period of 30 days. Clinical parameters such as gingival index, plaque index along with microbial colony forming units using plaque samples and antioxidant levels in saliva were estimated at baseline, 30 and 90 days’ time intervals.

**Results:**

All 3 groups showed gradual reduction in GI, PI and microbial counts. Considering the mean scores of recorded parameters at the scheduled time intervals, notable changes were observed between chlorhexidine and guava mouth rinse compared to placebo group. Although there was improvement in the antioxidant status in all study participants, yet there was no statistically significant difference observed.

**Conclusion:**

Guava mouth rinse can be used as an empirical adjunct to professional oral prophylaxis owing to its multifactorial properties and favourable acceptance. However, long term studies need to be conducted to validate its use for an extended period of time.

**Trial registration:**

The clinical trial has been prospectively registered on 17th February 2017 by the Clinical Trials Registry-India (CTRI/2017/02/007898).

## Background

Chronic gingivitis is widely accepted as the preliminary phase to a destructive process which gradually results in the loss of both soft and hard tissues surrounding the teeth. The dominant role of supragingival plaque control measures to curb this disease progression has been well established [1]. When appropriate oral hygiene techniques are practiced, there will be “effective” removal of plaque. However, a consistently effective form of tooth brushing and flossing may not be followed regularly, hence leaving behind residual plaque leading to gingival disease. Therefore, an additional chemotherapeutic approach has been recommended along with routine self-performed oral care practices [2]. Among them, mouth rinses has been the most frequently tested medium and consented for use globally. Chlorhexidine (CHX) mouthrinse have been universal since decades, however, owing to its varied side-effects (mainly staining and taste alteration), these are not recommended for a long term use [3]. This extended disproportionate use of synthetic antimicrobials and compounds have resulted in creating resistance and degrading its benefits. Hence, there has been a surge to adopt natural resources which has proven to be safe and effective for restoring normal health and above all cost-effective. Various randomized clinical trials [4–6] have been conducted and proven to exhibit the use of phytochemicals as an alternative therapy for management of systemic and dental diseases.

*Psidium guajava* L. is a fruit-bearing tree commonly known as “guava”, which belongs to the family, Myrtaceae. The leaves and bark of guava tree have an extensive record of medicinal uses. Many researchers have demonstrated the presence of a wide variety of bioactive compounds in the leaf of P.guajava that are capable of demonstrating valuable effects on human well-being. Literature have not provided conclusive evidence regarding potential side-effects of guava leaf extracts when taken in a dose-dependant manner. In general, phytochemical studies of P.guajava have revealed the presence of bioactive substances like tannins, triterpenes, flavonoids, essential and fixed oils, saponin, carotenoids, lectins, vitamins (C&A), alkaloids, glycosides, and reducing-sugars [7, 8]. Of these, flavonoids, is known for its antibacterial effect along with quercetin which is responsible for an anti-oxidant effect, which maintains the effectual functioning of the immune system [9]. The anti-inflammatory effect at a cellular level has been explained by Jang et al. wherein the authors found that guava leaf extract significantly impeded the lipopolysaccharide (LPS)-induced production of nitric oxide and prostaglandin E2 in a dose-dependent manner [10]. Considering its effect on local tissues, guava leaf extract has been used for apthous ulcers displaying a marked improvement in pain and resolution of ulcer within 7 days [11]. In view of literature references on valuable qualities of P.guajava, a preliminary investigation was carried out to check the efficacy of the leaf variants of P.guajava and based on these results, further research was carried out. Moreover,considering debased attributes to long term use of chlorhexidine mouthrinse, the present clinical trial was directed with a primary objective to study the antigingivitis and antiplaque effect of guava L based mouth rinse as an adjunct to scaling for management of chronic form of gingivitis. The secondary objective was to analyze the changes in microbial colony forming units (CFU) and antioxidant levels from baseline to 3 months.

The null hypothesis of this study is that, the additional use of guava L mouth rinse may be equally effective as 0.2% CHX mouth rinse in the reduction of clinical parameters (Gingival index, Plaque index).

## Methods

**Fig. 1:**
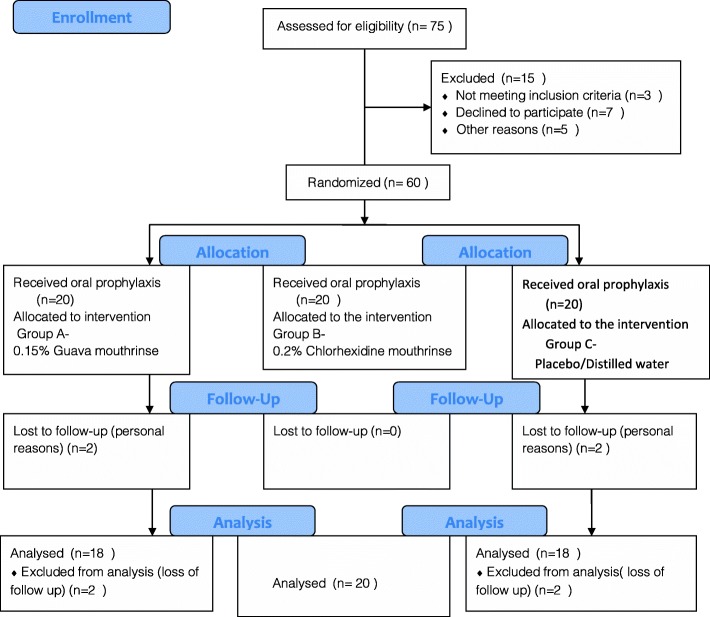
Flow chart depicting Consort statement

This clinical investigation was a double blind randomized, placebo controlled three-arm parallel design study involving subjects with moderate to severe chronic gingivitis. The study was approved by the Institutional ethics review board that was conducted in accordance with the helsinki declaration of 1975 revised in 2000. The present study followed the consort guidelines [12] (Fig. 1) and was registered by the clinical trial registry.

This study design comprised of primary section which included indigenous formulation of the mouthwash and an in vivo part which involved dispensing the formulated mouthrinse after professional oral prophylaxis and tabulating the pre and post outcomes.

### Plant materials

*Psidium guajava* leaves was collected from the Manipal Academy of Higher Education campus, Manipal, Karnataka, India during the months of June and July. The leaves of the guava plant was identified and authenticated by specialized personnel, Dr. Gopalakrishna Bhat, Professor of Botany &Taxonomy, Karnataka, India. A voucher specimen (specimen no. PP 620) was deposited in the Department of Pharmacognosy, Manipal College of Pharmaceutical Sciences, Manipal Academy of Higher Education, Manipal, Karnataka, India. After harvesting the leaves, it was thoroughly cleaned and dried and finely powdered.

**Fig. 2:**
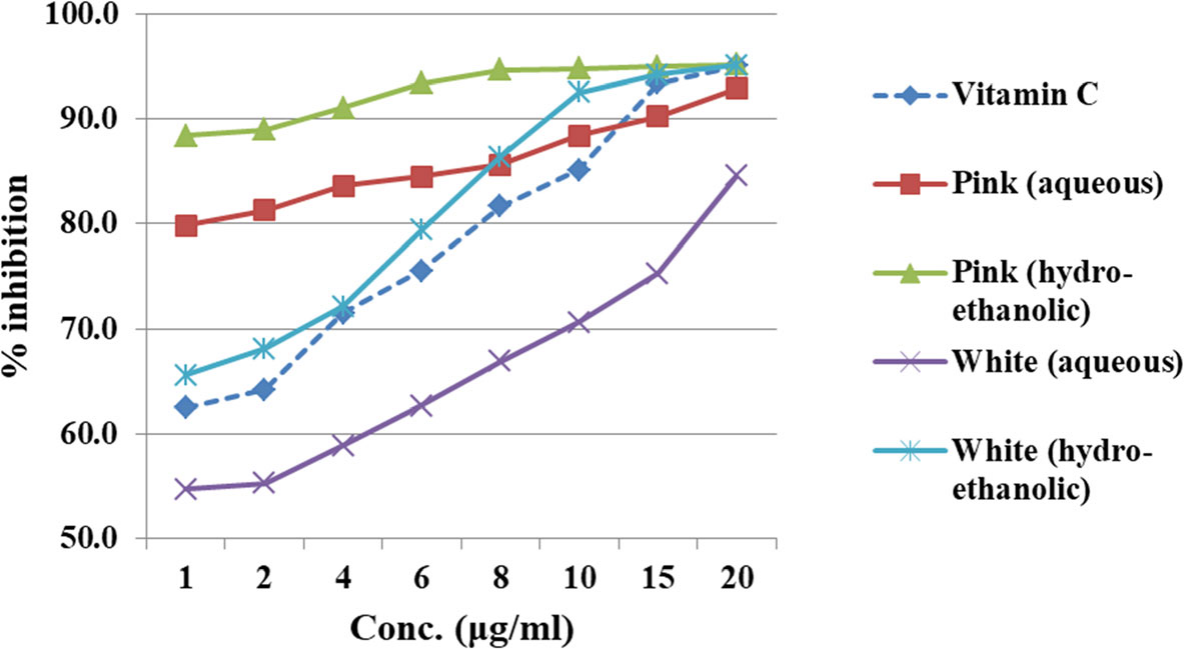
Ferric reducing power of standard and various formulations of guava leaf extract

**Fig. 3:**
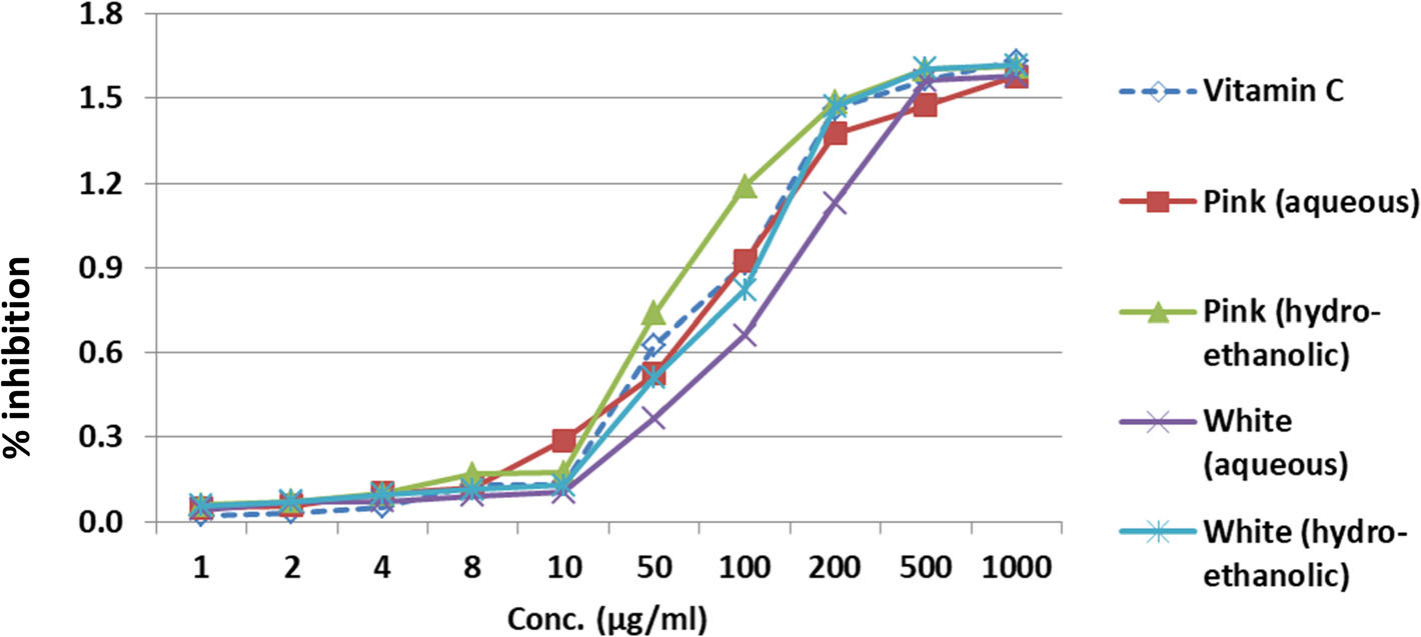
DPPH assay depicting free radical scavenging activity of standard ascorbic acid and Guava extract formulations

Considering the availability of guava fruit variants (i.e. the pink and the white variety), the antioxidant potential of these extracts were first studied using DPPH assay (2, 2-diphenyl 1–1-picrylhydrazyl) and Ferric reducing power assay (FRAP). Both aqueous and hydroethonolic extracts were prepared from the guava leaves of pink and white fruits. Following the results obtained from the assay (DPPH and FRAP), hydroethonolic extract of guava leaf (pink fruit variety) had better antioxidant property as compared to the white variety (Figs. 2 and 3). Thus, further part of the trial resulted in collection of guava leaves of efficacious pink fruit variety. The leaves were thoroughly washed, shade-dried at ambient temeperature. These dried leaves aids in stabilization of the biological material and increases its shelf life.

### Preparation of guava leaf extract

The withered guava leaves were ground to a coarse powder of approximately 600 g. The cold extraction or maceration procedure [13] was utilized for the preparation of guava extract. The powder was placed into a stoppered container with 70:30 hydro-ethanol (70% water and 30% alcohol) for 7 days, with frequent agitation, filtered and the marc was pressed. Following which the solvent was carefully evaporated in a vacuum evaporator (Rotavapor® R-100, Buchi, Mumbai, India) to obtain 50 g of dry extract.

### Determination of minimal inhibitory concentration (MIC)

Following the preparation of guava L extract, the MIC estimation was conducted in the Department of Microbiology, KMC, Manipal. Various dilutions (5 mg,10 mg, 15 mg, 20 mg) of the hydro-ethanolic extract was dissolved in 1 ml of dimethyl sulfoxide (solvent) to obtain concentrations of 0.05, 0.1,0.15 and 0.2%. All the concentrations were checked against the bacterial strains (S.mutans, S.oralis, S.mitis, A.viscosus, F.nucleatum). The MIC value of the extract was determined as the lowest concentration that completely inhibited bacterial growth after 48 h of incubation at 37 °C. An average value of 0.15% was taken for the formulation of the mouthrinse preparation.

### Preparation of guava leaf extract based mouthrinse

The oral rinse formulation of guava leaf extract was prepared by dissolving the accurately weighed dry guava leaf extract (0.15%) in absolute alcohol (1.1%) and homogenizing it with glycerol (15%), propylene glycol (15%), Tween 80 (1%). Further, menthol (0.1%) was added to this mixture, the remaining volume was amounted to 100% with distilled water. Absolute alcohol was used as a solvent to dissolve the extract and serves as a preservative. The shelf life of the mouth rinse was assessed by subjecting to accelerated stability studies as per ‘The International Council for Harmonisation of Technical Requirements for Pharmaceuticals for Human Use’ (ICH) guidelines (40 ± 2 °C/75 ± 5% RH) for 3 months. The mouthrinse in the storage container was stored in the stability chamber (Thermolab, Mumbai, India). The samples were withdrawn at different time intervals and analyzed for physical parameters and assay. No changes in physical appearance such as color, odor, consistency, and no phase separation was observed in mouthrinse during the stability study. The extracted content also remained unchanged as observed under UV analysis at 274 nm, indicating the stability of mouthrinse.

### In vivo clinical trial design and treatment protocol

The present clinical trial was a prospective, randomized placebo-controlled double blind parallel arm, interventional clinical study of 3-month duration. Patients who visited the out-patient unit of the department of Periodontology, Manipal College of dentals sciences, Karnataka, India from June 2017 to June 2018 were screened for the current clinical trial. Sixty patients who matched the enrolment criteria and willing to participate were qualified for this study. A signed informed consent was obtained prior to participation. Randomization was done using block size of 6 samples with an allocation ratio of 1:1. It was performed manually by using sequentially numbered opaque envelopes for the treatment allocation into 10 blocks with the each block containing 6 codes of samples.

The chief coordinator (JV) performed the initial examination, and randomly assigned the patients to one of three groups using the random allocation sequence. This examination was performed for patient recruitment. The groups assigned were Group A – 0.15% Guava mouth rinse; Group B – 0.2% Chlorhexidine mouth rinse (positive control) and Group C – Distilled water (placebo/negative control).

Inclusion criteria were as follows – (i) Presence of minimum 24 functional teeth (ii) Presence of chronic generalized moderate to severe form of gingivitis (iii) Patients within the age group of 18 to 45 years. Exclusion criteria were the following: systemically compromised patients; subjects with removable or fixed orthodontics appliance or prosthesis; subjects who were prescribed antibiotics or inflammatory drugs in the past 3 months; those on high polyphenol diet; those who had undergone periodontal therapy in previous 6 months; smoking or any form of tobacco; pregnant and lactating women; reported allergy to guava or chlorhexidine.

The clinician (NN), conducted oral examinations and scorings under similar working conditions at baseline, 1st month, 3rd month and also provided the patients with professional periodontal therapy. Mouth rinses were dispensed to the patients in identical amber coloured bottles with a code printed on it. Both the clinician and the patients were masked to the corresponding information.

The sequential steps involved during the initial visit of the participant included:

- Collection of supragingival plaque sample with a sterile site- specific gracey curette from the labial and/or buccal surfaces of teeth (mostly posterior teeth, since there is more prevalence of plaque accumulation) for the CFU estimation. The plaque samples were collected and transported in reduced transport fluid (RTF) and further analysed for CFU.

### Method of CFU estimation

Colony forming unit is an estimation of the number of viable bacterial cells in a given sample. The plaque samples were incubated for 48 h at 37 °C after which the specimens were diluted in normal saline (dilutions of 1:10, 1:100, 1:1000). Then, it was plated on blood agar and growth was observed on all inoculated plates. The average values were calculated as CFU/ml

b. Collection of saliva for estimation of the antioxidant activity using FRAP assay. For this purpose, the patients were asked to rinse the mouth and then expel the unstimulated saliva into a 5 ml sterile container which was then stored at -80 °C until use.
c. Recording of Clinical parameters [GI, PI]
  Gingival index (GI) proposed by Loe and Silness [14]- scoring criteria as follows; Score 0-normal gingiva: natural coral pink gingiva with no evidence of inflammation, Score 1-Mild inflammation: slight change in color,slight edema. No bleeding on probing. Score 2 –Moderate inflammation: redness, edema and glazing,bleeding on probing. Score 3 –Severe inflammation:marked redness and edema/ulceration/tendency to bleed spontaneously.

Turesky–Gilmore Glickman modification of Quigley Hein Plaque Index [15]. Scoring criteria is as follows;Score 0- no plaque, Score1- isolated areas of plaque at gingival margin, Score 2- Thin band of plaque at gingival margin(≤1 mm), Score 3- plaque covering upto 1/3 of the tooth surface, Score 4- plaque covering between 1/3 and 2/3 of the tooth surface, Score 5- plaque covering ≥2/3 of the tooth surface.

d. Subsequently, all the patients received professional oral prophylaxis which includes supragingival scaling, oral hygiene instructions and dispensing of the experimental mouth rinses with specific instructions regarding the dosage and directions for use. The patients were instructed to brush with a soft bristled toothbrush. Similar toothpastes were provided to all the patients by the examiner. 10 ml of mouth rinse with equal amount of dilution was instructed to be used twice daily,half an hour after toothbrushing for 1 min for a period of 30 days. The patients were asked to refrain from any other additional oral hygiene aids. The trial participants were instructed to return the mouthwash bottles at the time of 1 month follow-up visit to assess for compliance. During their visit, staining of the teeth and any irritation to the oral tissues were examined.

All the subjects were then recalled at intervals of 1 and 3 months for reevaluation of clinical parameters and also for collection of plaque and saliva samples for microbiological and biochemical analysis.

### Statistical analysis

To achieve 85% power (instituted by G*power, version 3.0.1; Franz Faul universitat, Kiel, Germany) and detect significant differences with effect size of 0.47 (*p* < 0.05), a total of 50 participants were required. To protect from possible drop outs, the sample size was increased to 60.

Normality assumption was checked using Shapiro-Wilk test and the data was observed to be not normally distributed. Hence, non parametric tests were applied. Friedman test was applied for intragroup comparison at different time intervals. The groups with statistical significance were then considered and Post hoc Wilcoxon Sign rank test was applied. Kruskal Wallis test was applied for Inter group comparison. Post hoc Mann Whitney test was applied to the groups which were significant for further comprehension.

## Results

The present study comprised of sixty patients aged between 18 and 40 years. Considering the gender distribution, group A had 12 males and 8 females, Group B: 11 males and 9 females and Group C: 12 males and 8 females accounting to a total of 35 males and 25 females paricipants. Although 60 patients enrolled initially for the study; only 56 patients completed the study and the rest 4 patients did not appear for the follow up intervals due to personal reasons. Hence the total number of patients were 18 in group A, 20 in group B and 18 in group C. No adverse events were reported by the participants during the course of the study.

### In vitro results

The results of MIC using various concentrations of guava leaf extract were tested on bacterial strains present in the oral cavity is summarized in Table 1.

**Table 1:** Concentrations of Guava L extract tested for bacterial strains commonly present in supragingival plaque

Sl. No.	Bacteria	5 mg/ml 0.05%	10 mg/ml 0.1%	15 mg/ml 0.15%	20 mg/ml 0.2%
1	*Streptococcus mutans*	R	S	S	S
2	*Streptococcus oralis*	R	R	S	S
3	*Streptococcus mitis*	R	R	S	S
4	*Actinomyces viscosus*	R	S	S	S
5	*Fusobacterium nucleatum*	R	R	S	S

*R* Resistant, *S* Sensitive

The results of DPPH assay and Ferric reducing assay exhibited that *hydroethonolic extract* of guava leaf (*pink fruit variety*) had better antioxidant property as compared to the white variety (Figs. 2 and 3).

### In vivo results

The descriptive data analysis considered in this present study among all the test groups at defined recall intervals are summarized in Table 2.

**Table 2:** Description of clinical parameters among the test groups at different time intervals

Groups	Time intervals	N	Minimum	Maximum	Mean ± Std. Deviation
GINGIVAL INDEX					
Group A	Baseline	20	1.10	2.64	1.86 ± 0.47
	1 month	18	.76	2.00	1.19 ± 0.34
	3 months	18	.20	1.45	.84 ± 0.34
Group B	Baseline	20	1.02	2.72	1.99 ± 0.47
	1 month	20	.57	2.10	1.29 ± 0.37
	3 months	20	.00	1.50	.62 ± 0.47
Group C	Baseline	20	1.32	2.72	2.21 ± 0.43
	1 month	19	.80	2.00	1.50 ± 0.40
	3 months	18	.70	1.50	1.23 ± 0.28
PLAQUE INDEX					
Group A	Baseline	20	3.00	4.00	3.15 ± 0.36
	1 month	18	1	3	2.06 ± 0.53
	3 months	18	1	2	1.71 ± 0.47
Group B	Baseline	20	2.00	4.00	3.20 ± 0.52
	1 month	20	1	2	1.8 ± 0.41
	3 months	20	1	2	1.60 ± 0.50
Group C	Baseline	20	3.00	5.00	3.65 ± 0.67
	1 month	19	2	3	2.44 ± 0.51
	3 months	18	2	2	2.36 ± 0.02
COLONY FORMING UNIT (*10^4^)					MEDIAN
Group A	Baseline	20	2	710	135
	1 month	18	.09	250.00	10.5
	3 months	18	.01	200.00	9
Group B	Baseline	20	0	670	125
	1 month	20	.01	530.00	21
	3 months	20	.00	510.00	8.05
Group C	Baseline	20	24	550	180
	1 month	19	7.10	510.00	120
	3 months	18	8.10	520.00	150
ANTI-OXIDANT LEVELS					MEDIAN
Group A	Baseline	20	.01	.85	0.109
	1 month	18	.08	.93	0.1905
	3 months	18	.00	1.26	0.4705
Group B	Baseline	20	.00	.97	0.094
	1 month	20	.00	1.45	0.2025
	3 months	20	.00	1.85	0.4525
Group C	Baseline	20	.06	1.42	0.139
	1 month	19	.04	1.23	0.125
	3 months	18	.05	1.66	0.4385

### Gingival status

Mean gingival scores of all the 3 test groups (A,B,C) at the scheduled time intervals were statistically analyzed (Table 2). Intergroup comparison using Kruskal wallis ANOVA test demonstrated statistically significant changes among all the groups at 1st and 3rd month recall interval (Table 3). Detailed analysis using post hoc Mann whitney test showed significant improvement between test group A and B compared to group C at the 3rd month recall interval (*p* = 0.006; *p* = 0.002) (Table 4). Intragroup comparison at different study time intervals demonstrated statistically significant difference from baseline to 3rd month recall time interval (Table 5).

**Table 3:** Comparison of the test groups using Kruskal-Wallis ANOVA test

		Group A^a^B	Group A ^a^C	Group B^a^C
Gingival index	1 month	0.11	0.02	0.18
	3 months	0.33	0.006^a^	0.002^a^
Plaque index	1 month	0.49	0.094	0.011^a^
	3 months	0.94	0.079	0.063
CFU	1 month	0.10	0.021	0.15
	3 months	0.86	0.005^a^	0.003^a^
Antioxidant levels	1 month	0.98	0.48	0.51
	3 months	0.62	0.38	0.23

^a^significant value set at 0.017(0.05/3 groups); Post hoc using Mann-whitney test

**Table 4:** Intergroup comparison of statistically significant results using Post hoc using Mann-whitney test

	GI	PI	CFU	Anti-oxidant levels
Baseline	0.072	0.11	0.69	0.13
1 month	0.04*	0.035*	0.036*	0.73
3 months	0.002*	0.14	0.004*	0.44

*significance (*p* < 0.05); Kruskal-Wallis ANOVA test

**Table 5:** Changes in the assessed parameters within the groups at different time intervals using Post hoc Wilcoxon sign rank test

		Baseline*1 month	Baseline*3 months	1 month * 3 months
Gingival index	Group A	0.00*	0.00*	0.00*
	Group B	0.00*	0.00*	0.00*
	Group C	0.00*	0.00*	0.001*
Plaque index	Group A	0.00*	0.00*	0.025
	Group B	0.00*	0.00*	0.046
	Group C	0.00*	0.00*	0.06
CFU	Group A	0.00*	0.00*	0.00*
	Group B	0.00*	0.00*	0.00*
	Group C	0.00*	0.00*	0.028*
Anti-oxidant levels	Group A	0.00*	0.00*	0.00*
	Group B	0.00*	0.00*	0.00*
	Group C	0.001*	0.00*	0.00*

### Plaque index

All the participants demonstrated high range of plaque scores at baseline which subsequently reduced at the recall time period (Table 2). Exploration of data exhibited statistically significant reduction in all the groups at the 1st month recall time period (*p* = 0.035) (Table 3). Intergroup comparison exhibited statistically significant difference between group B and C (*p* = 0.011) which was observed in 1st month recall interval (Table 4). Considering the observations demonstrated during the study period, there was a significant difference seen in all the test groups from baseline to 3rd month (Table 5).

### Microbial count estimation

Measurements related to colony forming units among the test groups showed reduction in microbial counts from baseline to 3rd month recall visit (Table 2). Comparative evaluation between the groups revealed statistically significant reduction in 1st and 3rd month recall period (*p* = 0.036; *p* = 0.004) (Table 3). However, at the end of 3rd month, statistically significant reduction in microbial colonies were seen between group A and B compared to group C (Table 4). Post hoc Wilcoxon sign rank test showed statistically significant difference between groups A, B and C at all time intervals (Table 5).

### Anti oxidant status

Oxidative stress present in saliva was assessed by FRAP assay and analyzed using UV spectrophotometry. All the subjects in the test groups exhibited enhanced antioxidant levels from baseline to 3rd month recall interval (Table 2). Detailed analysis between the test groups showed no statistically significant difference (Tables 3 and 4). However, taking into account the scheduled recall intervals, an improvement in the anti-oxidant status was observed (Table 5).

## Discussion

The obvious role of dental plaque as the etiological agent for gingivitis laid the basis for the importance of mechanical plaque control [16–18]. However, the pervasive occurrence of gingivitis often suggests that means to curb oral biofilms through mechanical measures still remains unsuccessful. Hence, adjunctive use of antimicrobial mouth rinses are effective and in demand as part of clinician’s prescription [19]. However, the long term use of chemotherapeutic agents have been opposed due to its undesirable outcome seeking more innocuous agents that can be used for extended time period. The experimental mouth rinse was compared to 0.2% chlorhexidine mouthrinse. The results of this 3 month clinical trial exhibited the notable role of 0.2% chlorhexidine mouth rinse at all scheduled study time intervals. However, the use of guava mouth rinse also displayed clinically comparable outcome in terms of maintenance of gingival health.

Initially the work commenced, with an exploration into the effectiveness of guava leaves (based on commercially available variants of white and pink fruit) incorporated in aqueous or hydroethanolic form. The observations from the antioxidant tests revealed that the hydroethanolic extract of pink guava leaf had superior activity and hence was used for the mouth rinse formulation (Figs. 2 and 3). Similar results based on efficacy of hydroethanolic extract of guava leaf was also stated by Jongkwon Seo et al. [20]

The present study had sixty trial participants, however, four patients were lost during the follow up interval. Patient compliance plays a major role for improvement and maintenance in oral health measures. Araújo MR and co-workers have provided strategic measures which could improve patient compliance after therapy to enhance clinical, behavioural and psychological parameters of periodontal health [21].

The present study was conducted on 56 subjects with moderate to severe form of gingivitis (GI mean score 2–3). All the participants showed reduction in the recorded clinical parameters during the course of the study period. (Tables 2 and 3). Such beneficial aftermath would have been facilitated by the effect of professional supragingival scaling in combination with standard tooth brushing technique (modified bass technique). This also be attributed to hawthrone effect. Similar conclusions were derived from the studies conducted by Hughes and Caffesse,1978 [22] and Suomi et al,1971 [23]. Considering the variances, in gingival index at scheduled time intervals it was observed that all the 3 groups exhibited statistically significant reduction, owing to the recall visits and reinforcement of oral hygiene instructions. Taking into account, intergroup comparison, it was observed that significant changes were reflected in the groups Guava and CHX at 3rd month compared to distilled water (placebo) group (Table 4). This could be explained by the superior action of chlorhexidine and guava mouthrinse in reducing gingival inflammation. This was in accordance to the study conducted by Grossman et al, 1986 [24] in which the authors found a reduction in gingival scores compared to placebo group. However, detailed data analysis demonstrated among the 3 groups, only the distilled water group did not exhibit significant decrease between the 1st to 3rd month (*p* = 0.021). This could be possibly explained by the initiation of re-occurrence of supragingival microbial flora to pretreatment levels, as stated in the literature review authored by Greenstein G,1992 [25].

Furthermore, the present study detected no statistical difference between guava and chlorhexidine mouth rinse groups at 1st and 3rd month interval (Table 4), suggesting similar functional activity between the two mouth rinses in lowering the GI scores which resulted in clinical improvements in both the experimental groups. This could be attributed to the presence of bioactive ingredients in guava, which contributed to its anti-inflammatory activity. Additionally, it has the ability to inhibit prostaglandins, kinin and histamines, which in turn substantiates the gingival health [26].

All the study subjects demonstrated improvement in plaque control during the study period with maximum significance observed in the CHX group at the 1st month recall period. These favorable results could be attributed to reinforcement of oral hygiene tooth brushing measures at recall visits, and the antiplaque property and substantive quality of chlorhexidine which led to the enhanced outcome. Similar observations were demonstrated by Pradeep et al, 2016 [5], comparing herbal mouth wash with CHX mouth rinse, their results exhibited the action of CHX was superior at all-time intervals of the study.

Likewise, guava mouth rinse group also showed beneficial results which matched with CHX mouth rinse both at 1st and 3rd month recall interval. Though the results were not statistically significant, the mean scores showed timely reductions in plaque scores and improvement in gingival health. These satisfactory effects can be accredited to the antimicrobial effect of guava due to the presence of its major ingredient present in leaf, guajaverin. Similar findings were correspondingly projected in the studies performed by Gomashe AV, 2014 and Prabhu GR, 2006 along with co-workers. The authors noticed the strong activity of guava against streptococcus mutans in the formation of dental biofilm [27, 28].

Assessment of microbial colony forming units in the present study was basically performed to quantify the amount of viable bacteria available on the tooth surface at each recall visit and to further study the antimicrobial property of the test mouth rinses. In the present study, Guava and CHX groups showed substantially significant reduction in CFU at all scheduled time intervals, except the placebo group which exhibited an increase in the microbial colonies suggesting the antimicrobial effect of both CHX and guava mouth rinse. An invitro study revealed the presence of glycosidal constituent, quercetin present in guava leaf to have noteworthy antibacterial action against periodontal pathogens which functions by disrupting the bacterial cell membrane and forming irreversible complexes by inactivating the extracellular proteins [29].

Taking into account the antioxidant status, the current study demonstrated statistically significant improvement in all test groups at the recall visits, which probably could be attributed to the professional prophylaxis rendered at baseline and reinforcement of oral hygiene instructions at recall intervals. Also, guava is known to have enriched levels of ascorbic acid, phenolic compounds (protocatechuic acid, ferulic acid, quercetin, guavin B and Gallic acid) which could have contributed to the improved anti-oxidant levels within the saliva. Additionally, it also possess the ability to scavenge free superoxide ions, hydrogen peroxide and prevent the formation of hydroxyl radical [30]. However,the present study did not exhibit statistically significant difference between the test groups. This could be reasoned by considering the fact that, the focused groups of study participants were chronic gingivitis wherein the severity of disease is less compared to periodontitis. These findings were comparable to the work conducted by Sudhakar et al 2015. The authors of the study analyzed the anti-oxidant status in saliva, plasma and GCF of patients with healthy periodontium, chronic gingivitis and chronic periodontitis. Their results showed oxidative stress to be increased in chronic periodonitits condition compared to other test groups [31].

### Novelty/public health significance/social relevance of this study

The current study was based on the formulation of an oral rinse developed from the leaves of a tropical fruit (guava) rich in high profile nutrients. The leaf extract of both the pink and white varieties of guava fruit were tested for its efficacy. Based on the results, guava leaf (pink fruit) extract was prepared for oral use so that the patients gain maximum benefit from the herbal product. In comparison to commercially available oral rinses containing chemical constituents (eg. Chlorhexidine) and its side effects on prolonged use, this adjunctive anti-plaque agent could be used safely to enhance healing.

### Limitation of the study

The limitation of the present study was the short term use of guava L extract oral rinse. This time slot was selected to compare with Chlorhexidine mouthrinse which is normally prescribed for a short period due to its marked side-effects.

## Conclusion

Guava leaf extract mouth rinse, resulted in benefits until the end of the study period which permits its utilization as an adjunct to professional oral prophylaxis. Although less potent compared to the chemical constituent (0.2% chlorhexidine mouthrinse), guava mouth rinse proved to have better antimicrobial property compared to placebo group. Hence, upholds its use has a favorable oral care product. An exploration into the limitations of CHX mouth rinse curbs its long term use. Therefore, guava mouth rinse can be enlisted among the phytotherapeutic alternatives for maintaining healthy gingiva. Nevertheless, further randomized clinical trials should be carried out for an extended time period to substantiate its long term effects.

## Data Availability

All the data obtained and materials analyzed in this research are available with the corresponding author upon request.

## Abbreviations

CFU: Colony forming units
CHX: Chlorhexidine
DPPH: Assay (2, 2-diphenyl 1–1-picrylhydrazyl
FRAP: Ferric reducing power assay
GI: Gingival index
MIC: Minimum inhibitory concentration
PI: Plaque Index
